# Penta­terbium lithium tris­tannide, Tb_5_LiSn_3_

**DOI:** 10.1107/S1600536811041328

**Published:** 2011-10-12

**Authors:** Andrij Stetskiv, Ivan Tarasiuk, Renata Misztal, Volodymyr Pavlyuk

**Affiliations:** a Ivano-Frankivsk National Medical University, Department of Chemistry, Galytska str. 2, 76018 Ivano-Frankivsk, Ukraine; b Department of Inorganic Chemistry, Ivan Franko Lviv National University, Kyryla and Mefodiya str., 6, 79005 Lviv, Ukraine; c Institute of Chemistry, Environment Protection and Biotechnology, Jan Dlugosz University, al. Armii Krajowej 13/15, 42-200 Czestochowa, Poland

## Abstract

The new ternary phase penta­terbium lithium tris­tannide, Tb_5_LiSn_3_, crystallizes in the hexa­gonal Hf_5_CuSn_3_ structure type, which is a ‘filled’ version of the binary *RE*_5_Sn_3_ phases (Mn_5_Si_3_-type) (*RE* is rare earth). The asymmetric unit contains two Tb sites (site symmetries 3.2 and *m*2*m*), one Li site (site symmetry 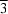.*m*) and one Sn site (site symmetry *m*2*m*). The 14-vertex Frank–Kasper polyhedra are typical for Li and Tb atoms. The environment of the Sn atom is a pseudo-Frank–Kasper polyhedron with a coordination number of 13 for the tin atom. One of the Tb atoms is enclosed in a 17-vertex polyhedron. The metallic type of bonding was indicated by an analysis of the inter­atomic distances.

## Related literature

For the Hf_5_CuSn_3_ structure type, see: Rieger & Parthé (1965 ▶). For related structures, see: Pavlyuk & Bodak (1992*a* ▶,*b* ▶); Pavlyuk *et al.* (1989 ▶, 1991 ▶, 1993 ▶). For the magnetic properties of related compounds, see: Tran *et al.* (2008 ▶).

## Experimental

### 

#### Crystal data

Tb_5_LiSn_3_
*M* *_r_* = 1157.72
Hexagonal, 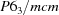

*a* = 9.0122 (14) Å
*c* = 6.5744 (13) Å
*V* = 462.4 (2) Å^3^
*Z* = 2
Mo *K*α radiation
μ = 45.56 mm^−1^
*T* = 293 K
0.07 × 0.05 × 0.03 mm

#### Data collection

Oxford Diffraction Xcalibur3 CCD diffractometer
Absorption correction: analytical (*CrysAlis RED*; Oxford Diffraction, 2008 ▶) *T*_min_ = 0.322, *T*_max_ = 0.657
1907 measured reflections
216 independent reflections
207 reflections with *I* > 2σ(*I*)
*R*_int_ = 0.021

#### Refinement

*R*[*F* ^2^ > 2σ(*F* ^2^)] = 0.021
*wR*(*F* ^2^) = 0.066
*S* = 1.33
216 reflections
14 parameters
Δρ_max_ = 1.08 e Å^−3^
Δρ_min_ = −1.47 e Å^−3^

### 

Data collection: *CrysAlis CCD* (Oxford Diffraction, 2008 ▶); cell refinement: *CrysAlis CCD*; data reduction: *CrysAlis RED* (Oxford Diffraction, 2008 ▶); program(s) used to solve structure: *SHELXS97* (Sheldrick, 2008 ▶); program(s) used to refine structure: *SHELXL97* (Sheldrick, 2008 ▶); molecular graphics: *DIAMOND* (Brandenburg, 2006 ▶); software used to prepare material for publication: *SHELXL97*.

## Supplementary Material

Crystal structure: contains datablock(s) I, global. DOI: 10.1107/S1600536811041328/ff2031sup1.cif

Structure factors: contains datablock(s) I. DOI: 10.1107/S1600536811041328/ff2031Isup2.hkl

Additional supplementary materials:  crystallographic information; 3D view; checkCIF report

## Figures and Tables

**Fig. 1.**
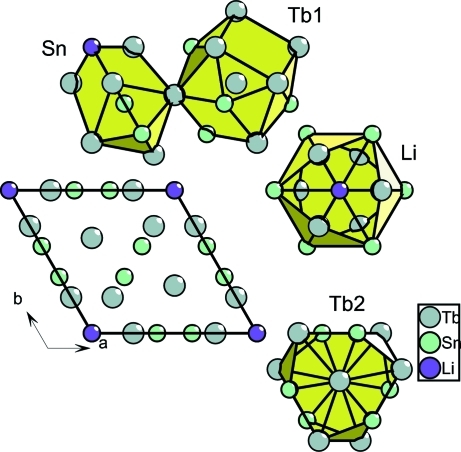
The projection of the unit cell and coordination polyhedra of the atoms.

**Fig. 2.**
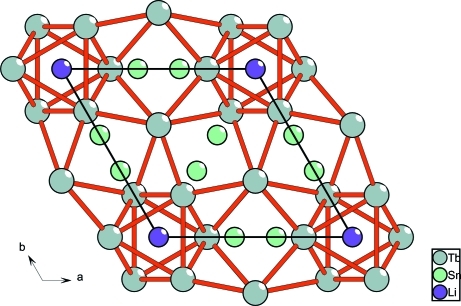
The distribution of tin and lithium atoms in three-dimensional-nets consisted of Tb atoms.

## supplementary crystallographic information

### Comment

The RE_5_T*M*_3_ (RE - rare earth, T - Cu, Ag and *M* - Sn, Pb) ternary stannides crystallize in a hexagonal Hf_5_CuSn_3_ (superstructure to Ti_5_Ga_4_-type) with space group *P*6_3_*/mcm* (Rieger and Parthé, 1965). These intermetallic compounds are characterized by two different sites for the RE atoms located at *4 d* and *6 g*, respectively. The Sn or Pb atoms are located at the next *6 g* site and the transitions atoms occupy *2 b* site.The RE_5_T*M*_3_ intermetallics are 'filled' version of the binary RE_5_*M*_3_ phases which crystallize in Mn_5_Si_3_ structure type. It is also possible, that the transition metals fill the octahedral voids.

For the Ce based compounds, Ce_5_T*M*_3_, investigated by (Tran *et al.*, 2008) are found multiple magnetic phase transitions at low temperatures and discussed the role of *f-spd* hybridization on the evolution of heavy-fermion behaviour.

We detected the new ternary compound during the systematic study of ternary alloys of Tb—Li—Sn system from the concentration region with low content of lithium. The powder diffraction pattern of this compound is similar to the powder pattern of the RE_5_Sn_3_ (RE - rare-earth metals) binary phases, but has some differences. So we decided to further study this phase using single-crystal method. Obtained single-crystal data show that the title compound crystallizes with the hexagonal space group *P*6_3_/*mcm* as a Hf_5_CuSn_3_ type. The projection of the unit cell and coordination polyhedra of the atoms are shown in Fig. 1. The distribution of tin and lithium atoms in three-dimensional-nets consisted of Tb atoms are shown in Fig. 2.

The number of neighbouring atoms correlates well with the dimensions of the central atoms. The Tb atoms are enclosed in 14- and 17-vertex polyhedra. The coordination polyhedron of the Sn atom is pseudo Frank-Kasper polyhedron with CN=13. Lithium atom is surrounded by 14 neighbours atoms in the form of 14-vertex Frank-Kasper polyhedron. The shortest interatomic distances in the title compound are in the typical for intermetallic compounds ranges and indicate metallic type of bonding.

In the title compound lithium atoms occupy the same crystallographic position that the atoms of transition metal in the original structure type. The same was observed previously when we studied RELiSn_2_ compounds with the CeNiSi_2_ structure type (Pavlyuk *et al.*, 1989), RELiGe with the ZrNiAl type (Pavlyuk *et al.*, 1991 and Pavlyuk & Bodak, 1992*a*), RE_3_Li_2_Ge_3_ with Hf_3_Ni_2_Si_3_ type (Pavlyuk & Bodak, 1992*b*), solid solutions RLi*_x_*Cu_2 -_*_x_*Si_2_ and RLi*_x_*Cu_2 -_*_x_*Ge_2_ (Pavlyuk *et al.*, 1993).

### Experimental

Terbium, lithium and tin, all with a nominal purity more than 99.9 wt. %, were used as starting elements. First, the pieces of the pure metals with a stoichiometry Tb_55_Li_10_Sn_35_ were pressed into pellet, enclosed in tantalum crucible and placed in a resistance furnace with a thermocouple controller. Heating rate from room temperature to 670 K was equal 5 K per minute. At this temperature the alloy was held over 2 d and then the temperature was increased from 670 to 1070 K over 1 h. Then the alloy was annealed at this temperature for 8 h and slowly cooled down to room temperature. After the melting and annealing procedures, the total weight loss was less than 2%. Small good quality single-crystal of the title compound was isolated from alloy.

### Crystal data

**Table:**

Tb_5_LiSn_3_	*D*_x_ = 8.315 Mg m^−^^3^
*M**_r_* = 1157.72	Mo *K*α radiation, λ = 0.71073 Å
Hexagonal, *P*6_3_/*m**c**m*	Cell parameters from 1907 reflections
Hall symbol: -P 6c 2	θ = 2.6–27.4°
*a* = 9.0122 (14) Å	µ = 45.56 mm^−^^1^
*c* = 6.5744 (13) Å	*T* = 293 K
*V* = 462.4 (2) Å^3^	Prism, metallic dark grey
*Z* = 2	0.07 × 0.05 × 0.03 mm
*F*(000) = 956	

### Data collection

**Table:**

Oxford Diffraction Xcalibur3 CCD diffractometer	216 independent reflections
Radiation source: fine-focus sealed tube	207 reflections with *I* > 2σ(*I*)
graphite	*R*_int_ = 0.021
Detector resolution: 0 pixels mm^-1^	θ_max_ = 27.4°, θ_min_ = 2.6°
ω scans	*h* = −11→11
Absorption correction: analytical (*CrysAlis RED*; Oxford Diffraction, 2008)	*k* = −11→11
*T*_min_ = 0.322, *T*_max_ = 0.657	*l* = 0→8
1907 measured reflections	

### Refinement

**Table:**

Refinement on *F*^2^	0 restraints
Least-squares matrix: full	Primary atom site location: structure-invariant direct methods
*R*[*F*^2^ > 2σ(*F*^2^)] = 0.021	Secondary atom site location: difference Fourier map
*wR*(*F*^2^) = 0.066	*w* = 1/[σ^2^(*F*_o_^2^) + (0.0268*P*)^2^ + 3.977*P*] where *P* = (*F*_o_^2^ + 2*F*_c_^2^)/3
*S* = 1.33	(Δ/σ)_max_ < 0.001
216 reflections	Δρ_max_ = 1.08 e Å^−^^3^
14 parameters	Δρ_min_ = −1.47 e Å^−^^3^

### Special details

**Table:**

Geometry. All e.s.d.'s (except the e.s.d. in the dihedral angle between two l.s. planes) are estimated using the full covariance matrix. The cell e.s.d.'s are taken into account individually in the estimation of e.s.d.'s in distances, angles and torsion angles; correlations between e.s.d.'s in cell parameters are only used when they are defined by crystal symmetry. An approximate (isotropic) treatment of cell e.s.d.'s is used for estimating e.s.d.'s involving l.s. planes.
Refinement. Refinement of *F*^2^ against ALL reflections. The weighted *R*-factor *wR* and goodness of fit *S* are based on *F*^2^, conventional *R*-factors *R* are based on *F*, with *F* set to zero for negative *F*^2^. The threshold expression of *F*^2^ > σ(*F*^2^) is used only for calculating *R*-factors(gt) *etc*. and is not relevant to the choice of reflections for refinement. *R*-factors based on *F*^2^ are statistically about twice as large as those based on *F*, and *R*- factors based on ALL data will be even larger.

### Fractional atomic coordinates and isotropic or equivalent isotropic displacement parameters (Å^2^)

**Table:**

	*x*	*y*	*z*	*U*_iso_*/*U*_eq_	
Tb1	0.25088 (10)	0.0000	0.2500	0.0479 (3)	
Tb2	0.3333	0.6667	0.0000	0.0535 (3)	
Sn3	0.60694 (14)	0.0000	0.2500	0.0493 (4)	
Li4	0.0000	0.0000	0.0000	0.055 (13)	

### Atomic displacement parameters (Å^2^)

**Table:**

	*U*^11^	*U*^22^	*U*^33^	*U*^12^	*U*^13^	*U*^23^
Tb1	0.0478 (4)	0.0481 (5)	0.0479 (5)	0.0241 (3)	0.000	0.000
Tb2	0.0536 (4)	0.0536 (4)	0.0532 (6)	0.0268 (2)	0.000	0.000
Sn3	0.0491 (5)	0.0493 (7)	0.0496 (6)	0.0247 (4)	0.000	0.000
Li4	0.07 (2)	0.07 (2)	0.03 (2)	0.033 (11)	0.000	0.000

### Geometric parameters (Å, °)

**Table:**

Tb1—Li4	2.7952 (8)	Tb2—Tb1^xii^	3.8093 (7)
Tb1—Li4^i^	2.7952 (8)	Tb2—Tb1^xi^	3.8093 (7)
Tb1—Sn3^ii^	3.1066 (11)	Tb2—Tb1^xiii^	3.8093 (8)
Tb1—Sn3^iii^	3.1066 (11)	Tb2—Tb1^ix^	3.8093 (7)
Tb1—Sn3	3.2090 (16)	Sn3—Tb1^xvii^	3.1066 (11)
Tb1—Sn3^iv^	3.5281 (8)	Sn3—Tb1^xviii^	3.1066 (11)
Tb1—Sn3^v^	3.5281 (8)	Sn3—Tb2^vii^	3.2247 (6)
Tb1—Tb2^vi^	3.8093 (7)	Sn3—Tb2^vi^	3.2247 (6)
Tb1—Tb2^vii^	3.8093 (7)	Sn3—Tb2^ix^	3.2247 (6)
Tb1—Tb2^viii^	3.8093 (7)	Sn3—Tb2^viii^	3.2247 (6)
Tb1—Tb2^ix^	3.8093 (7)	Sn3—Tb1^iv^	3.5281 (8)
Tb1—Tb1^x^	3.9161 (17)	Sn3—Tb1^v^	3.5281 (8)
Tb2—Sn3^xi^	3.2247 (6)	Li4—Tb1^xix^	2.7952 (8)
Tb2—Sn3^xii^	3.2247 (6)	Li4—Tb1^xx^	2.7952 (8)
Tb2—Sn3^xiii^	3.2247 (6)	Li4—Tb1^xiii^	2.7952 (8)
Tb2—Sn3^ix^	3.2247 (6)	Li4—Tb1^x^	2.7952 (8)
Tb2—Sn3^xiv^	3.2247 (6)	Li4—Tb1^xiv^	2.7952 (8)
Tb2—Sn3^iii^	3.2247 (6)	Li4—Li4^i^	3.2872 (6)
Tb2—Tb2^xv^	3.2872 (6)	Li4—Li4^xxi^	3.2872 (6)
Tb2—Tb2^xvi^	3.2872 (6)		
			
Li4—Tb1—Li4^i^	72.03 (3)	Sn3^xiv^—Tb2—Tb1^xii^	144.94 (2)
Li4—Tb1—Sn3^ii^	82.67 (2)	Sn3^iii^—Tb2—Tb1^xii^	123.785 (13)
Li4^i^—Tb1—Sn3^ii^	82.67 (2)	Tb2^xv^—Tb2—Tb1^xii^	64.439 (7)
Li4—Tb1—Sn3^iii^	82.67 (2)	Tb2^xvi^—Tb2—Tb1^xii^	115.561 (7)
Li4^i^—Tb1—Sn3^iii^	82.67 (2)	Sn3^xi^—Tb2—Tb1^xi^	53.50 (2)
Sn3^ii^—Tb1—Sn3^iii^	161.86 (5)	Sn3^xii^—Tb2—Tb1^xi^	110.39 (2)
Li4—Tb1—Sn3	143.985 (13)	Sn3^xiii^—Tb2—Tb1^xi^	59.520 (16)
Li4^i^—Tb1—Sn3	143.985 (13)	Sn3^ix^—Tb2—Tb1^xi^	51.60 (2)
Sn3^ii^—Tb1—Sn3	99.07 (2)	Sn3^xiv^—Tb2—Tb1^xi^	123.785 (13)
Sn3^iii^—Tb1—Sn3	99.07 (2)	Sn3^iii^—Tb2—Tb1^xi^	144.94 (2)
Li4—Tb1—Sn3^iv^	147.31 (3)	Tb2^xv^—Tb2—Tb1^xi^	115.561 (7)
Li4^i^—Tb1—Sn3^iv^	75.28 (2)	Tb2^xvi^—Tb2—Tb1^xi^	64.439 (7)
Sn3^ii^—Tb1—Sn3^iv^	93.283 (5)	Tb1^xii^—Tb2—Tb1^xi^	63.16 (2)
Sn3^iii^—Tb1—Sn3^iv^	93.283 (5)	Sn3^xi^—Tb2—Tb1^xiii^	59.520 (16)
Sn3—Tb1—Sn3^iv^	68.70 (3)	Sn3^xii^—Tb2—Tb1^xiii^	123.785 (13)
Li4—Tb1—Sn3^v^	75.28 (2)	Sn3^xiii^—Tb2—Tb1^xiii^	53.50 (2)
Li4^i^—Tb1—Sn3^v^	147.31 (3)	Sn3^ix^—Tb2—Tb1^xiii^	144.94 (2)
Sn3^ii^—Tb1—Sn3^v^	93.283 (5)	Sn3^xiv^—Tb2—Tb1^xiii^	110.39 (2)
Sn3^iii^—Tb1—Sn3^v^	93.283 (5)	Sn3^iii^—Tb2—Tb1^xiii^	51.60 (2)
Sn3—Tb1—Sn3^v^	68.70 (3)	Tb2^xv^—Tb2—Tb1^xiii^	64.439 (7)
Sn3^iv^—Tb1—Sn3^v^	137.41 (5)	Tb2^xvi^—Tb2—Tb1^xiii^	115.561 (7)
Li4—Tb1—Tb2^vi^	102.887 (9)	Tb1^xii^—Tb2—Tb1^xiii^	102.753 (8)
Li4^i^—Tb1—Tb2^vi^	136.924 (8)	Tb1^xi^—Tb2—Tb1^xiii^	93.848 (16)
Sn3^ii^—Tb1—Tb2^vi^	54.444 (14)	Sn3^xi^—Tb2—Tb1^ix^	123.785 (13)
Sn3^iii^—Tb1—Tb2^vi^	140.12 (2)	Sn3^xii^—Tb2—Tb1^ix^	59.520 (16)
Sn3—Tb1—Tb2^vi^	53.886 (12)	Sn3^xiii^—Tb2—Tb1^ix^	144.94 (2)
Sn3^iv^—Tb1—Tb2^vi^	100.83 (2)	Sn3^ix^—Tb2—Tb1^ix^	53.50 (2)
Sn3^v^—Tb1—Tb2^vi^	51.970 (13)	Sn3^xiv^—Tb2—Tb1^ix^	51.60 (2)
Li4—Tb1—Tb2^vii^	136.924 (8)	Sn3^iii^—Tb2—Tb1^ix^	110.39 (2)
Li4^i^—Tb1—Tb2^vii^	102.887 (9)	Tb2^xv^—Tb2—Tb1^ix^	115.561 (7)
Sn3^ii^—Tb1—Tb2^vii^	140.12 (2)	Tb2^xvi^—Tb2—Tb1^ix^	64.439 (7)
Sn3^iii^—Tb1—Tb2^vii^	54.444 (14)	Tb1^xii^—Tb2—Tb1^ix^	93.848 (16)
Sn3—Tb1—Tb2^vii^	53.886 (12)	Tb1^xi^—Tb2—Tb1^ix^	102.753 (8)
Sn3^iv^—Tb1—Tb2^vii^	51.970 (13)	Tb1^xiii^—Tb2—Tb1^ix^	160.55 (2)
Sn3^v^—Tb1—Tb2^vii^	100.83 (2)	Tb1^xvii^—Sn3—Tb1^xviii^	78.14 (5)
Tb2^vi^—Tb1—Tb2^vii^	107.77 (2)	Tb1^xvii^—Sn3—Tb1	140.93 (2)
Li4—Tb1—Tb2^viii^	136.924 (8)	Tb1^xviii^—Sn3—Tb1	140.93 (2)
Li4^i^—Tb1—Tb2^viii^	102.887 (9)	Tb1^xvii^—Sn3—Tb2^vii^	73.951 (16)
Sn3^ii^—Tb1—Tb2^viii^	54.444 (14)	Tb1^xviii^—Sn3—Tb2^vii^	137.78 (3)
Sn3^iii^—Tb1—Tb2^viii^	140.12 (2)	Tb1—Sn3—Tb2^vii^	72.61 (2)
Sn3—Tb1—Tb2^viii^	53.886 (12)	Tb1^xvii^—Sn3—Tb2^vi^	137.78 (3)
Sn3^iv^—Tb1—Tb2^viii^	51.970 (13)	Tb1^xviii^—Sn3—Tb2^vi^	73.951 (16)
Sn3^v^—Tb1—Tb2^viii^	100.83 (2)	Tb1—Sn3—Tb2^vi^	72.61 (2)
Tb2^vi^—Tb1—Tb2^viii^	51.122 (14)	Tb2^vii^—Sn3—Tb2^vi^	145.22 (4)
Tb2^vii^—Tb1—Tb2^viii^	86.152 (16)	Tb1^xvii^—Sn3—Tb2^ix^	73.951 (16)
Li4—Tb1—Tb2^ix^	102.887 (9)	Tb1^xviii^—Sn3—Tb2^ix^	137.78 (3)
Li4^i^—Tb1—Tb2^ix^	136.924 (8)	Tb1—Sn3—Tb2^ix^	72.61 (2)
Sn3^ii^—Tb1—Tb2^ix^	140.12 (2)	Tb2^vii^—Sn3—Tb2^ix^	61.287 (15)
Sn3^iii^—Tb1—Tb2^ix^	54.444 (14)	Tb2^vi^—Sn3—Tb2^ix^	107.56 (2)
Sn3—Tb1—Tb2^ix^	53.886 (12)	Tb1^xvii^—Sn3—Tb2^viii^	137.78 (3)
Sn3^iv^—Tb1—Tb2^ix^	100.83 (2)	Tb1^xviii^—Sn3—Tb2^viii^	73.951 (16)
Sn3^v^—Tb1—Tb2^ix^	51.970 (13)	Tb1—Sn3—Tb2^viii^	72.61 (2)
Tb2^vi^—Tb1—Tb2^ix^	86.152 (16)	Tb2^vii^—Sn3—Tb2^viii^	107.56 (2)
Tb2^vii^—Tb1—Tb2^ix^	51.122 (14)	Tb2^vi^—Sn3—Tb2^viii^	61.287 (15)
Tb2^viii^—Tb1—Tb2^ix^	107.77 (2)	Tb2^ix^—Sn3—Tb2^viii^	145.22 (4)
Li4—Tb1—Tb1^x^	45.533 (9)	Tb1^xvii^—Sn3—Tb1^iv^	73.62 (2)
Li4^i^—Tb1—Tb1^x^	45.533 (9)	Tb1^xviii^—Sn3—Tb1^iv^	73.62 (2)
Sn3^ii^—Tb1—Tb1^x^	50.93 (2)	Tb1—Sn3—Tb1^iv^	111.30 (3)
Sn3^iii^—Tb1—Tb1^x^	110.93 (2)	Tb2^vii^—Sn3—Tb1^iv^	68.510 (11)
Sn3—Tb1—Tb1^x^	150.0	Tb2^vi^—Sn3—Tb1^iv^	125.693 (6)
Sn3^iv^—Tb1—Tb1^x^	108.33 (2)	Tb2^ix^—Sn3—Tb1^iv^	125.693 (6)
Sn3^v^—Tb1—Tb1^x^	108.33 (2)	Tb2^viii^—Sn3—Tb1^iv^	68.510 (11)
Tb2^vi^—Tb1—Tb1^x^	99.726 (11)	Tb1^xvii^—Sn3—Tb1^v^	73.62 (2)
Tb2^vii^—Tb1—Tb1^x^	148.420 (11)	Tb1^xviii^—Sn3—Tb1^v^	73.62 (2)
Tb2^viii^—Tb1—Tb1^x^	99.726 (11)	Tb1—Sn3—Tb1^v^	111.30 (3)
Tb2^ix^—Tb1—Tb1^x^	148.420 (11)	Tb2^vii^—Sn3—Tb1^v^	125.693 (6)
Sn3^xi^—Tb2—Sn3^xii^	163.38 (4)	Tb2^vi^—Sn3—Tb1^v^	68.510 (11)
Sn3^xi^—Tb2—Sn3^xiii^	72.44 (2)	Tb2^ix^—Sn3—Tb1^v^	68.510 (11)
Sn3^xii^—Tb2—Sn3^xiii^	96.334 (10)	Tb2^viii^—Sn3—Tb1^v^	125.693 (6)
Sn3^xi^—Tb2—Sn3^ix^	96.334 (10)	Tb1^iv^—Sn3—Tb1^v^	137.41 (5)
Sn3^xii^—Tb2—Sn3^ix^	72.44 (2)	Tb1^xix^—Li4—Tb1^xx^	88.933 (19)
Sn3^xiii^—Tb2—Sn3^ix^	97.06 (4)	Tb1^xix^—Li4—Tb1	91.067 (19)
Sn3^xi^—Tb2—Sn3^xiv^	96.334 (10)	Tb1^xx^—Li4—Tb1	180.0
Sn3^xii^—Tb2—Sn3^xiv^	97.06 (4)	Tb1^xix^—Li4—Tb1^xiii^	180.0
Sn3^xiii^—Tb2—Sn3^xiv^	163.38 (4)	Tb1^xx^—Li4—Tb1^xiii^	91.067 (19)
Sn3^ix^—Tb2—Sn3^xiv^	96.334 (10)	Tb1—Li4—Tb1^xiii^	88.933 (19)
Sn3^xi^—Tb2—Sn3^iii^	97.06 (4)	Tb1^xix^—Li4—Tb1^x^	91.067 (19)
Sn3^xii^—Tb2—Sn3^iii^	96.334 (10)	Tb1^xx^—Li4—Tb1^x^	91.067 (19)
Sn3^xiii^—Tb2—Sn3^iii^	96.334 (10)	Tb1—Li4—Tb1^x^	88.933 (19)
Sn3^ix^—Tb2—Sn3^iii^	163.38 (4)	Tb1^xiii^—Li4—Tb1^x^	88.933 (19)
Sn3^xiv^—Tb2—Sn3^iii^	72.44 (2)	Tb1^xix^—Li4—Tb1^xiv^	88.933 (19)
Sn3^xi^—Tb2—Tb2^xv^	120.643 (7)	Tb1^xx^—Li4—Tb1^xiv^	88.933 (19)
Sn3^xii^—Tb2—Tb2^xv^	59.357 (8)	Tb1—Li4—Tb1^xiv^	91.067 (19)
Sn3^xiii^—Tb2—Tb2^xv^	59.357 (7)	Tb1^xiii^—Li4—Tb1^xiv^	91.067 (19)
Sn3^ix^—Tb2—Tb2^xv^	120.643 (8)	Tb1^x^—Li4—Tb1^xiv^	180.00 (7)
Sn3^xiv^—Tb2—Tb2^xv^	120.643 (7)	Tb1^xix^—Li4—Li4^i^	126.015 (13)
Sn3^iii^—Tb2—Tb2^xv^	59.357 (7)	Tb1^xx^—Li4—Li4^i^	126.015 (13)
Sn3^xi^—Tb2—Tb2^xvi^	59.357 (7)	Tb1—Li4—Li4^i^	53.985 (13)
Sn3^xii^—Tb2—Tb2^xvi^	120.643 (7)	Tb1^xiii^—Li4—Li4^i^	53.985 (13)
Sn3^xiii^—Tb2—Tb2^xvi^	120.643 (8)	Tb1^x^—Li4—Li4^i^	53.985 (13)
Sn3^ix^—Tb2—Tb2^xvi^	59.357 (8)	Tb1^xiv^—Li4—Li4^i^	126.015 (13)
Sn3^xiv^—Tb2—Tb2^xvi^	59.357 (7)	Tb1^xix^—Li4—Li4^xxi^	53.985 (13)
Sn3^iii^—Tb2—Tb2^xvi^	120.643 (7)	Tb1^xx^—Li4—Li4^xxi^	53.985 (13)
Tb2^xv^—Tb2—Tb2^xvi^	180.0	Tb1—Li4—Li4^xxi^	126.015 (13)
Sn3^xi^—Tb2—Tb1^xii^	110.39 (2)	Tb1^xiii^—Li4—Li4^xxi^	126.015 (13)
Sn3^xii^—Tb2—Tb1^xii^	53.50 (2)	Tb1^x^—Li4—Li4^xxi^	126.015 (13)
Sn3^xiii^—Tb2—Tb1^xii^	51.60 (2)	Tb1^xiv^—Li4—Li4^xxi^	53.985 (13)
Sn3^ix^—Tb2—Tb1^xii^	59.520 (16)	Li4^i^—Li4—Li4^xxi^	180.0

Symmetry codes: (i) −*x*, −*y*, *z*+1/2; (ii) −*y*, *x*−*y*−1, *z*; (iii) −*x*+*y*+1, −*x*+1, *z*; (iv) −*x*+1, −*y*, −*z*+1; (v) −*x*+1, −*y*, −*z*; (vi) *x*, *y*−1, *z*; (vii) −*x*+1, −*y*+1, *z*+1/2; (viii) *x*, *y*−1, −*z*+1/2; (ix) −*x*+1, −*y*+1, −*z*; (x) −*x*+*y*, −*x*, *z*; (xi) *y*, −*x*+*y*+1, −*z*; (xii) *x*, *y*+1, *z*; (xiii) −*y*, *x*−*y*, *z*; (xiv) *x*−*y*, *x*, −*z*; (xv) *x*, *y*, −*z*+1/2; (xvi) *x*, *y*, −*z*−1/2; (xvii) −*y*+1, *x*−*y*, *z*; (xviii) −*x*+*y*+1, −*x*, *z*; (xix) *y*, −*x*+*y*, −*z*; (xx) −*x*, −*y*, −*z*; (xxi) −*x*, −*y*, *z*−1/2.
